# Use of the Assessment of Caregiver Experience with Neuromuscular Disease (ACEND) in Spinal Muscular Atrophy

**DOI:** 10.3390/jcm13040921

**Published:** 2024-02-06

**Authors:** Laurey Brown, Katie Hoffman, Chiara Corbo-Galli, Siyuan Dong, Katelyn Zumpf, Christa Weigel, Colleen Blomgren, Hannah Munson, Jessa Bidwell, Vamshi Rao, Nancy L. Kuntz, Abigail Schwaede, Kristin J. Krosschell

**Affiliations:** 1 Department of Rehabilitation Services, Ann & Robert H. Lurie Children’s Hospital of Chicago, Chicago, IL 60611, USA; khoffman@lureichildrens.org (K.H.); cmblomgren@luriechildrens.org (C.B.); 2 Division of Neurology, Ann & Robert H. Lurie Children’s Hospital of Chicago, Chicago, IL 60611, USAvrao@luriechildrens.org (V.R.); nkuntz@luriechildrens.org (N.L.K.); aschwaede@luriechildrens.org (A.S.); 3 Weinberg College of Arts and Sciences, Northwestern University, Evanston, IL 60208, USA; chiaracorbo-galli2025@u.northwestern.edu; 4 Department of Preventive Medicine, Division of Biostatistics, Northwestern University Feinberg School of Medicine, Chicago, IL 60611, USA; siyuan.dong@northwestern.edu (S.D.);; 5 Department of Pediatrics, Northwestern University Feinberg School of Medicine, Chicago, IL 60611, USA; k-krosschell@northwestern.edu; 6 Department of Physical Therapy and Human Movement Sciences, Northwestern University Feinberg School of Medicine, Chicago, IL 60611, USA

**Keywords:** spinal muscular atrophy, caregiver burden, quality of life, neuromuscular, pediatric, ACEND

## Abstract

**Background**: Spinal muscular atrophy (SMA) has a remarkable impact on function and participation. Subsequently, the caregivers of individuals with SMA are impacted as well. Providers and the SMA community should be aware of the presence of and likely expectations for the existence of caregiver burden. **Methods**: The Assessment of Caregiver Experience with Neuromuscular Disease (ACEND) quantifies caregivers’ perceptions of function and quality of life pertaining to time, finance and emotion. Analyses were conducted among SMA types and ambulatory and ventilatory status. Participants with SMA had varying ranges of function and were on pharmaceutical treatment. Total ACEND score, longitudinal change in total ACEND score, total quality of life (QOL) score, change in total QOL score and subdomains for QOL, including time, emotion and finance, were all explored. **Results**: Overall, the ACEND demonstrated discriminant validity and some observed trends. Total ACEND scores improved for caregivers of those with SMA 2, remained stable longitudinally for caregivers of those with SMA 1 and 3 and were not influenced by ventilation status. The caregivers of individuals with SMA 1 had the lowest total quality of life (QOL) score, as did the caregivers of non-ambulatory individuals and those requiring assisted ventilation. Longitudinally, there were no changes in total QOL between caregivers of individuals with different SMA types or ambulatory or ventilation status. There were some differences in emotional needs, but no differences in financial impact between the caregivers of individuals with different types of SMA or ambulatory and ventilatory status. **Conclusions**: With this information enlightening the presence of caregiver burden and expected changes in burden with pharmaceutical treatment, providers, third party payors and the SMA community at large can better assist, equip and empower those providing the necessary assistance to enable the lives of those with SMA.

## 1. Introduction

Spinal muscular atrophy (SMA) is a hereditary neurodegenerative disease leading to the degradation of motor neurons with resultant muscle weakness. The global impact of this disease has significant implications on individuals’ health and function, as well as repercussions for their caregivers and families. Natural history studies have historically focused on function, with less attention paid to concerns related to the individuals and the caregivers’ quality of life (QOL). The World Health Organization defines QOL as an individual’s perception of their situation in life in the context of culture, values, goals, expectations, standards and concerns [1]. Knowledge of the impact of caregiver QOL is necessary as it assists and educates providers in understanding real-world experience, enlightens third party payors to the needs encountered when providing care and informs the greater community of potential expectations and impact. Knowledge of the specific parameters that have the greatest impact on caregiver QOL can be a focus for improvement aimed at reducing strain and challenges. Influencers of QOL are multifactorial.

While some studies have looked at QOL as related to the individual [2,3,4], Xu et al. [5] found that the caregiver and affected individual’s QOL can differ significantly and that assessing the caregiver’s QOL, as captured by the ACEND, can be much more complex and multidimensional. There are often many elements to consider, including psychological make-up, coping strategies, patient and caregiver temperaments and the presence or absence of daily, social and economic support structures. One way to consider these elements of quality of life is to subcategorize them based on finance, time and emotion.

Farrar et al. [6] looked more specifically at the financial impact on families caring for a child with SMA and found that caregivers often must balance the costs of care and the allocation of resources. Additional concerns regarding financial challenges have been highlighted by Pacione et al. [7], with media reports citing concerns pertaining to insurance coverage.

The budgeting of time is an additional consideration to address, with Farrar et al. [6] finding that families have decreased opportunity for both leisure and social interactions, with accessibility being a key limiting factor [6]. Another study looking at individual and parent perspectives on nusinersen highlighted added concerns related to time. These included the length of time for drug approval, time and burden required to receive regular injections and manage side effects and the time constraints reflected in the lifelong commitment to drug treatment [8]. 

Finally, caregiver emotional well-being is another contributor to QOL due to the concerns of mental fatigue, stress and increased worry about financial burden [9]. Initial difficulties in receiving diagnosis, including the confrontation for potential of premature death and the impending loss of functional ability, have also been highlighted. These difficulties also involve the need to make difficult treatment choices, come to terms with lost expectations and limitations on social activities and the potential for increasing isolation [10].

There are multiple available tools used to assess the varied domains affecting individual QOL, with no best practice consensus. General tools may not accurately depict caregiver experience or the extent of specificity for neuromuscular disease. Selected tools need to assess the primary areas of concern and be sensitive to changes in function and presentation over time. 

For our study, the Assessment of Caregiver Experience with Neuromuscular Disease (ACEND) was used. In clinical trials, this scale applied in combination with functional rating scales, such as the Hammersmith Functional Motor Scale Expanded (HFMSE) and the Revised Upper Limb Module (RULM), to provide information that is sensitive to change and correlated with function. The ACEND is a caregiver impact-based reported outcome measure that has demonstrated reliability for responsiveness to the burden of care when evaluating individuals with cerebral palsy [5] and has been noted to be a valid and disease-specific scale for assessing and quantifying the experience of caregivers of individuals with neuromuscular disease [11]. It has also been found to be helpful in assessing perceived quality of life [5]. Additionally, the ACEND has been applied to the SMA 2 and 3 population to look at the impact of ongoing nusinersen treatment, with the correlate of health-related quality of life for caregivers [8].

This study aims to assess the validity of the ACEND for demonstrating caregiver perception of functional level ability and tracking further changes with treatment. With this knowledge, we can demonstrate the consistency of caregiver perception as assessed on a standardized measure. It also assesses the application of the ACEND to quantify the quality of life of the caregivers of individuals with different types of SMA and ambulatory and ventilation status. Finally, it seeks to determine if there are any longitudinal caregiver QOL changes with treatment between individuals with different types of SMA or ventilation or ambulatory status.

## 2. Materials and Methods

### 2.1. Ethics Statement

This natural history study was approved by the Institutional Review Board (IRB) at Ann and Robert H. Lurie Children’s Hospital of Chicago (LCH). Before inclusion in the natural history study, all individuals or their parent(s)/legal guardian(s) provided written informed consent and/or assent in accordance with local IRB guidelines. Research was conducted in accordance with the Helsinki Declaration of 1975. Data were deidentified for analysis.

### 2.2. Participants

All individuals with SMA enrolled in this natural history study at LCH received commercial treatment with nusinersen or onasemnogene abeparvovec-xioi and had a caregiver complete the ACEND on three or more visits from 02 November 2016 to 27 August 2019. These two pharmaceutical treatments were the only FDA-approved options at the time of data collection. The caregiver accompanying the patient to the appointment completed the survey, but the consistency of caregiver was not tracked for each visit. For the purpose of this paper, we applied the historic definitions of SMA types, as grouped by highest level of function achieved: SMA 1—unable to sit independently; SMA 2—able to sit but not walk independently; SMA 3—able to walk although not necessarily able to maintain this skill throughout lifespan [2]. By way of additional analysis, participants were also grouped by ambulatory and ventilatory status. Ambulatory was defined as the ability to walk without an assistive device for ≥15 m.

### 2.3. Procedures and Outcomes

Each individual’s age, SMA type and ambulatory and ventilation status were recorded on each visit. The ACEND questionnaire was provided to the parent/caregiver, using the approved written Spanish translation when indicated. The parent/caregiver accompanying the child completed the questionnaire and indicated their relationship to the patient in the study. Additionally, appropriate motor function measures (based on functional status and age) were completed, including the Children’s Hospital of Philadelphia—Infant Test of Neuromuscular Disorders (CHOP-INTEND), Hammersmith Functional Motor Scale Expanded (HFMSE) and Revised Upper Limb Module (RULM). For patients receiving nusinersen, data were collected prospectively within the 2-week window prior to baseline and at each follow-up visit (Days 60, 180, 300, 420, 500, 660, 780, 900 and 1020) once they had enrolled. For patients receiving onasemnogene abeparvovec-xioi, data were collected at baseline and at each follow-up visit, which included 1 month, 12 weeks, 6 months, 1 year and annually following the completion of the first year. 

Surveys were completed using a pen and paper with experienced physical therapists providing brief instructions on the completion of the assessment. Assistance in clarifying questions, when needed, was offered. Caregivers were advised not to calculate the section totals and to consider their perspective of the patient’s ability and function when completing the measure.

### 2.4. Outcome Measures

#### ACEND

The ACEND instrument includes 2 domains, 7 subdomains and 41 items. Domain 1 examines physical impact, including the 4 subdomains of feeding/grooming/dressing (6 items), sitting/play (5 items), transfers (5 items) and mobility (7 items). Domain 2 examines general caregiver impact, including 3 subdomains: time (4 items), emotion (9 items) and finance (5 items) [11]. For items in Domain 1, scores range from N/A to 6, with a score of 6 demonstrating the highest level of function. For items in Domain 2, scores range from 1–5, with a score of 5 demonstrating the lowest impact on quality of life. The total score is the sum of Domain 1 and Domain 2 with the highest achievable scores [5]. Therefore, a higher score is indicative of caregivers experiencing less impact in the areas of finance, time and emotion when providing care [11].

The time subdomain takes into consideration time for social events, caring for family members, partner/spouse time and vacations. The emotion subdomain considers feelings regarding the child’s health condition, stress or sadness regarding the condition, stress around interpersonal relationships, family tensions, limitations and interruptions to family activities, interruptions to attending events and parties, feelings of being trapped/stuck and worry about pain/discomfort. The finance subdomain considers the costs of medical appointments, the impact of pharmaceutical interventions, the impact of extra expenditures, the impact of travel expenses and the loss of household income.

In its initial validation and development, the ACEND was shown to have an overall relevance rating of 6.21+/−0.37 and a clarity rating of 6.68+/−0.52 (scale 0–7). Item total/item correlation (r = 0.4/0.5) demonstrated item validity (Cronbach alpha). Content validity showed that most caregivers believed the items to be important. Criterion validity was indicated by the significant differences in total and subdomain scores (and impact health) across GMFCS levels. Construct validity was considered acceptable and demonstrated by floor and ceiling effects of <40%. There were multiple floor effects for motor-based questions but no floor or ceiling effects for Domain 2 [11].

### 2.5. Standardized Motor Assessments 

The CHOP-INTEND, HFMSE and RULM were all administered in this study by trained physical therapists who utilized previously reported standardized procedures [12,13,14]. All tests required ~15 min each to perform, minimal participant burden and standardized equipment. All tests have demonstrated reliability and validity and are widely used in clinical trials for SMA to document natural history and disease trajectories. They have also demonstrated reliability, validity and sensitivity in clinical trials for nusinersen and onasemnogene abeparvovec-xioi [12,13,14,15].

#### 2.5.1. CHOP-INTEND

CHOP-INTEND is a disease-specific gross motor function measure that consists of 16 items assessing gross motor function in individuals with SMA 1 [12]. It is based on a total score of 64, with both left and right sides ranked and the best side used for the total score. The CHOP-INTEND was administered to all non-sitting participants.

#### 2.5.2. HFMS-E 

The HFMS-E is a disease-specific gross motor function measure that consists of 33 items assessing gross motor function in individuals with SMA 2 and 3 [13,14]. Scores are based on 0, 1 or 2, with a highest achievable total score of 66. The HFMS-E was administered to all sitters and walkers who were older than 2 years of age.

#### 2.5.3. RULM

The RULM is a disease-specific measure utilized to assess upper limb function for individuals with SMA across the functional spectrum. For the purposes of this study, the test was administered to individuals that were able to maintain an upright sitting position either independently or with the support of chair (wheelchair or otherwise) and were over 3 years of age [12]. The highest achievable score on the RULM is 37. It was administered and scored on both sides. The total RULM score was then calculated, which was a conglomerate of the performance on both sides.

### 2.6. Statistical Methods

Descriptive statistics were used to characterize the caregivers and participants with SMA. Frequency and percentage were recorded for the categorical variables and the means, standard deviations, medians and ranges for numeric variables. 

For this analysis, Domain 2 of the ACEND was used to quantify caregiver quality of life, with separate analyses for the specific Domain 2 subdomains of time, emotion and finance. To determine whether total or subdomain ACEND scores changed longitudinally, a linear mixed-effects regression was performed, including a random intercept to account for repeated measurements. Fixed effects included age at baseline, BMI measured at each visit and the number of years since initial treatment. To evaluate whether changes in ACEND scores over time were different between caregivers with experiences that were potentially impacted by SMA type or ambulatory or ventilatory status, a group-by-time interaction and corresponding main effects were added singularly into the model. The total ACEND score was also utilized to determine whether there was a correlation between an individuals’ functional motor performance, as measured by the CHOP-INTEND, HFMSE and RULM, and impact on the caregiver. 

Analyses were performed using R (version 4.2.2) and SAS, with an assumed 5% level of significance, unless otherwise specified. There were no adjustments for multiple hypothesis testing. Correlations were first made based on SMA type. Further analyses were conducted to see whether the specific functional covariates of ambulatory and ventilation status had specific impact on caregiver perspectives.

## 3. Results

In total, 34 participants with SMA were assessed over a period of at least two years. Of these 34, 9 were classified with SMA 1, 13 with SMA 2 and 12 with SMA 3. Additionally, four of the individuals were ventilated and seven were ambulatory. The mean age for all participants was 79.3 months. The mean age for individuals with SMA 1 was 53.3 months, SMA 2 was 82.1 months and SMA 3 was 95.8 months. The mean age for those on ventilation was 96.3 months and for those that were ambulatory, it was 76.8 months. There was a total of 32 mothers and 16 fathers who completed the survey. This number was different from total participants as, on occasion, a different caregiver would accompany the child. See Table 1 for detailed demographics.

### 3.1. Type Comparison (Figure 1)

#### 3.1.1. Total Longitudinal Score

The longitudinal trend of caregivers’ total ACEND scores was noted to be different between types (F = 4.64; *p*-value = 0.0111). Longitudinal data from the caregivers of participants with SMA 2 created a slope that was 10.30 points greater than that for caregivers of those with SMA 3 (B: 10.30; 95% CI (3.27, 17.32); *p*-value = 0.0044). There was an increase of 11.27 points/year for the caregivers of those with SMA 2 (11.27; 95% CI (6.53, 16.00); *p*-value < 0.0001). This increase is likely indicative of reduced caregiver burden. No differences were noted in the trends of total ACEND scores between the caregivers of those with SMA 1 and SMA 3.

**Figure 1:**
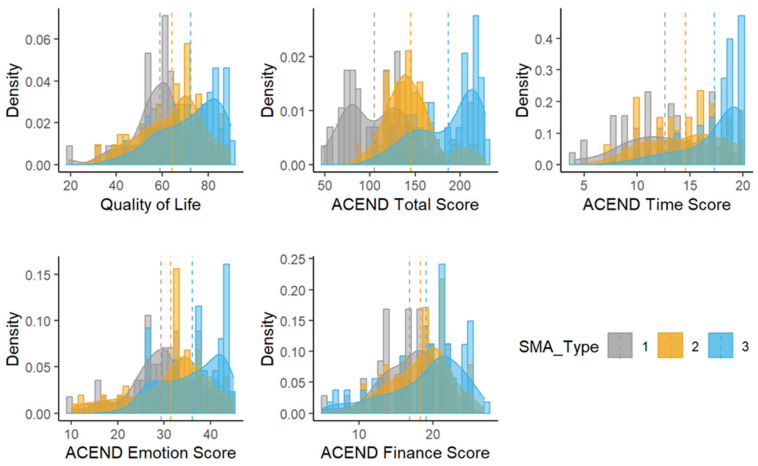
Histograms of outcomes by SMA type according to the Assessment of Caregiver Experience with Neuromuscular Disease (ACEND). hashed line: median for type.

#### 3.1.2. Time Subdomain

The ACEND time subtest score was 5.70 points lower on average among the caregivers of participants with SMA 1 compared to the caregivers of those with SMA 3 (B: −5.70; 95% CI: (−8.34, −3.07); *p*-value = 0.0001). The caregivers of those with SMA 2 had ACEND time subtest scores that were 2.70 points lower on average than the caregivers of those with SMA 3 (B: −2.70; 95% CI (−4.97, −0.43); *p*-value = 0.0214).

The longitudinal slopes and changes in ACEND time subtest scores did not differ between the caregivers of different SMA types, even after the model was simplified by removing the time*SMA type interaction (B: 0.45; 95% CI (−0.01, 0.92); *p*-value = 0.0569).

#### 3.1.3. Emotion Subdomain

The ACEND emotion scores were different between the caregivers of participants with SMA 1 and SMA 3, with the emotion scores for caregivers of those with SMA 1 being 9.40 points lower compared to that of the caregivers of those with SMA 3 (B: −9.40; 95% CI (−14.7, −4.07); *p*-value = 0.0011). There was no evidence that there were any differences in ACEND emotion scores between the caregivers of those with SMA 2 and 3 (B: −4.59; 95% CI (−9.19, 0.01); *p*-value = 0.0505).

As demonstrated in the ACEND time scores, there was not enough evidence to support longitudinal changes in caregivers’ ACEND emotion scores; thus, a simplified model was presented by removing the time-by-SMA type interaction. Once simplified, there was no suggestion of longitudinal differences between caregiver groups in terms of ACEND emotion scores (B: −0.17; 95% CI (−1.10, 0.77); *p*-value = 0.7251).

#### 3.1.4. Finance Subdomain

Similar to the time and emotion subdomains, differences in the finance subdomain scores did not appear significant between the caregivers of those with different SMA types (F = 0.14; *p*-value = 0.8713). After removing the time-by-SMA type interaction, there were no changes in longitudinal finance scores (B:0.43; 95% CI (−0.19, 1.04); *p*-value = 0.1712), nor were there differences between types (F = 1.47; *p*-value = 0.2464).

#### 3.1.5. Quality of Life (Domain 2: TIME + EMOTION + FINANCE)

When the three subdomains of time, emotion and finance were added to create the overall quality of life (QOL) quotient, there was a difference when comparing the caregivers of participants with SMA 1 and SMA 3. Specifically, QOL scores were 18.5 points lower among the caregivers of those with SMA 1 compared to the caregivers of those with SMA 3 (B: −18.5; 95% CI (−28.6, −8.34); *p*-value = 0.0008). There were no differences in total quality of life between the caregivers of participants with SMA 2 and 3 (B: −8.16; 95% CI (−16.9, 0.55); *p*-value = 0.0654).

There were no longitudinal changes in QOL scores for the caregivers of individuals with different SMA types.

### 3.2. Ambulatory Status Comparison (Figure 2)

#### 3.2.1. Total Score

The total ACEND score was 39.5 points lower on average among the caregivers of those that were non-ambulatory (B: −39.5; 95% CI (−53.9, −25.1); *p*-value < 0.0001). For both ambulatory and non-ambulatory individuals, the total caregiver ACEND score increased by 3.96 points per year on average (B:3.96; 95% CI (0.76, 7.16); *p*-value = 0.0156). The changes in total ACEND scores did not appear to differ over time between the caregivers of those that could and could not walk (F = 0.06; *p*-value = 0.8050).

**Figure 2:**
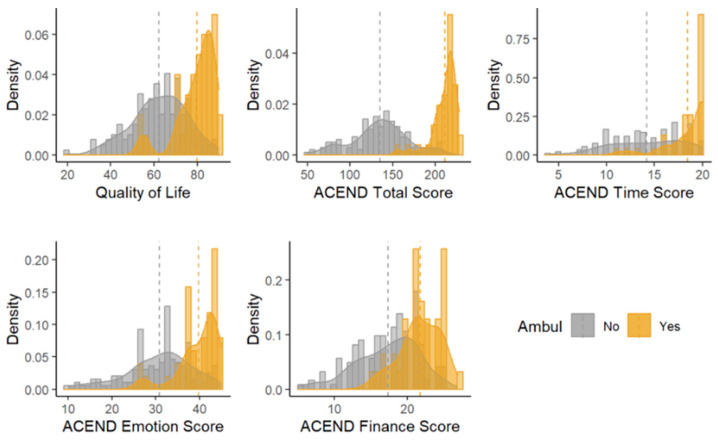
Histograms of outcomes by ambulatory status according to the Assessment of Caregiver Experience with Neuromuscular Disease (ACEND). hashed line: median for type.

#### 3.2.2. Time Subdomain

The changes in time scores for caregivers did not differ based on ambulatory status (F = 0.26; *p*-value = 0.6098). On average, there was no linear longitudinal trend in ACEND time scores (B:0.31; 95% CI (−0.17, 0.79); *p*-value = 0.2006) and there did not appear to be any differences in ACEND time scores between the caregivers of those with varied ambulatory status (F = 3.85; *p*-value = 0.0523).

#### 3.2.3. Emotion Subdomain

The ACEND emotion score was 5.09 points lower among the caregivers of those who were non-ambulatory (B: −5.09; 95% CI (−8.70, −1.48); *p*-value = 0.0062) when age at baseline, BMI and time were constant. Therefore, caregiver emotion scores were constant from baseline throughout the time points, with non-ambulatory status being consistently lower. The changes in ACEND emotion scores did not differ by ambulatory status and there was no linear longitudinal trend in emotion scores.

#### 3.2.4. Finance Subdomain

The caregivers of non-ambulatory individuals with SMA had finance scores 2.58 lower than the caregivers of ambulatory individuals (B: −2.58; 95% CI (−4.82, −0.35); *p*-value = 0.0239). Longitudinally, finance scores did not differ by ambulatory status.

#### 3.2.5. Quality of Life (Domain 2: TIME + EMOTION + FINANCE)

Total QOL scores were 8.99 points lower for the caregivers of individuals who were non-ambulatory (B = −8.99; 95% CI (−15.6, −2.41); *p*-value = 0.0079). The QOL scores did not differ longitudinally for the entire group of SMA caregivers, nor when stratified by the children’s ambulatory status (F = 0.65; *p*-value = 0.4221).

### 3.3. Ventilation Status Comparison (Figure 3)

#### 3.3.1. Total Score

The total ACEND score at baseline was 82.28 points higher for the caregivers of those that did not require ventilation compared to the caregivers of those that did require ventilation (B: 82.28; 95% CI (40.90, 123.7); *p*-value = 0.0003). On average, the total caregiver ACEND scores for both ventilated and non-ventilated participants increased 5.64 points every year.

**Figure 3:**
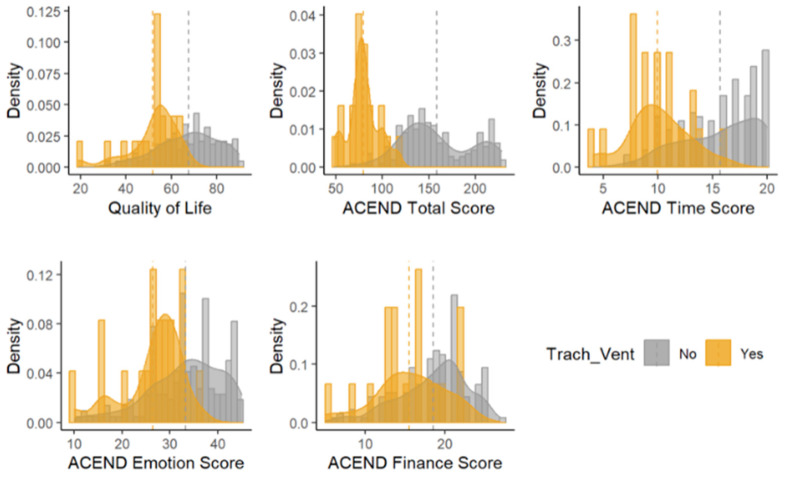
Histograms of outcomes by ventilation status according to the Assessment of Caregiver Experience with Neuromuscular Disease (ACEND).

#### 3.3.2. Time Subdomain

For the time subdomain scores, the slope was 2.12 points lower among the caregivers of those without ventilation (B: −2.12; 95% CI (−4.03, −0.21); *p*-value = 0.0295) and there was not enough evidence to suggest that ACEND time subdomain scores changed longitudinally (B: 0.15; 95% CI (−0.31, 0.61); *p*-value = 0.5180). For the caregivers of individuals requiring ventilation, the ACEND time scores increased by 2.27 points each year, on average (B: 2.27; 95% CI (0.41, 4.14); *p*-value = 0.0173).

#### 3.3.3. Emotion Subdomain

The ACEND emotion scores did not change longitudinally for the caregivers of those with or without ventilation (B: −0.33; 95% CI (−1.29, 0.63); *p*-value = 0.4943) and there was not enough evidence to suggest that the emotion scores differed by ventilation status (B: 5.51; 95% CI (−0.88, 11.90); *p*-value = 0.0897).

#### 3.3.4. Finance Subdomain

A longitudinal difference was noted in changes in ACEND finance scores between the caregivers of those that were ventilated and those that were not (F = 5.53; *p*-value = 0.0199). Specifically, the slope of the finance scores was 3.01 points lower among the caregivers of those that were not ventilated (B = −3.01; 95% CI (−5.54, −0.48); *p*-value = 0.0199). Longitudinally, finance scores were expected to increase by 3.15 points per year for the caregivers of participants requiring ventilation (B: 3.15; 95% CI (0.69, 5.62); *p*-value = 0.0125). There were not enough data to suggest that ACEND finance scores changed longitudinally for the caregivers of those without ventilation (B = 0.14; 95% CI (−0.48, 0.76); *p*-value = 0.6511).

#### 3.3.5. Quality of Life (Domain 2: TIME + EMOTION + FINANCE)

The QOL score was 14.47 points higher among the caregivers of those that were not ventilated (B: 14.47; 95% CI (1.31, 27.63); *p*-value = 0.0322). The changes in Domain 2 or quality of life scores were not significant longitudinally and did not differ between the caregivers of those with and without ventilation (F = 3.49; *p*-value = 0.0634).

### 3.4. Total ACEND Score as Related to Function

The correlation between total caregiver ACEND scores and scores on the CHOP, HFMS-E and RULM was moderate-to-strong for each type of SMA. For the CHOP, there was a Pearson correlation of 0.76, 0.88 for the HFMS-E and 0.77 for the RULM. There was a demonstrated *p*-value of <0.0001 for all measures. There were no correlations between caregiver QOL and scores on the standardized assessments (Table 2).

## 4. Discussion

There is a need to establish and standardize assessments that characterize and quantify caregiver quality of life as related to the perceived function of individuals with a specific neuromuscular disease. Furthermore, due to the varying functional levels of individuals with SMA, there needs to be an assessment tool that depicts functional ability, considers perspectives in changes in function over time and further examines potential caregiver QOL correlations. Once caregiver quality of life is appropriately measured, true needs can be fully identified. The impact of treatment on an individual’s functional performance and on caregiver quality of life is also important to consider. Since the advent of disease-targeted treatments for SMA, impacts on caregivers have not been fully studied. The use of real-world data, such as those applied in our study, has been shown to offer insights that help to enlighten changing natural history, advance the standard of care and bridge the gap between research and real life. Such studies have noted the potential value of obtaining information on the quality of everyday lives, as we strived to do in our study [16].

In this study, the use of the total ACEND score yielded expected differences between SMA types, with the caregivers of SMA 1 individuals demonstrating the lowest scores and the caregivers of SMA 3 individuals demonstrating the highest scores. Similar trends in total scores for those who were ambulatory versus non-ambulatory and those who were ventilated versus not ventilated were also demonstrated. This measure, therefore, has discriminant validity across SMA types and functional groups. In contrast to the analysis of the use of the ACEND with CP, there were no noted floor effects for participants with lower functional abilities or ceiling effects for those with mid-range abilities [11].

The total ACEND score was used to look at longitudinal trends across the caregivers of those with different SMA types, ambulatory status and ventilatory status. When looking at the longitudinal changes in total scores, there were improvements among tcaregivers of those with SMA 2 and stable scores for the caregivers of those with SMA 1 and SMA 3. This suggests that, longitudinally, the caregivers of individuals with SMA 2 may experience decreased impact and burden compared to the caregivers of individuals with SMA 1 and 3 where the impact is likely to remain stable. This finding may demonstrate consistency between caregivers and individuals in that for individuals, mostly with SMA 2, there is a preference to receive nusinersen, based on its potential to slow or stop disease progression and subsequently improve QOL while maintaining independence [7].

Additionally, both for the caregivers of ambulatory and non-ambulatory individuals, there was a longitudinal increase in the total ACEND score. This suggests a general improvement with treatment. While the caregivers of individuals not requiring ventilatory support had the expected higher total scores than the caregivers of individuals requiring ventilation, these scores increased in both groups at the same rate over time. Therefore, non-ventilated status was not associated with an improved rate of change and there were no associations for treatment on ventilatory status. 

These total scores help to provide valuable insights into what caregivers could expect when providing care to individuals with varying types of SMA, as well as differing ambulatory and ventilatory statuses. It provides a sense of caregiver perspective on function, as well as changes in function over time. Given these results, providers may be able to set clearer expectations for what could occur with treatment.

This study utilized Domain 2 of the ACEND, with a conglomerate score of time, emotion and finance, to quantify total caregiver QOL. When looking at total caregiver QOL scores, the caregivers of individuals with SMA 1 demonstrated the lowest scores, followed by those of individuals with SMA 2, while the caregivers of individuals with SMA 3 had the highest total QOL scores. The caregivers of non-ambulatory individuals had lower total QOL scores, as did the caregivers of individuals who required ventilation. With treatment, there were no changes in total caregiver QOL for individuals with different types of SMA or ambulatory status. However, there was a demonstrated trend of improved QOL over time for the caregivers of individuals requiring ventilatory support. 

It is often presumed that there is an association between QOL and a person’s functional ability, with milder phenotypes having improved QOL. While this has not been proven true in adult studies, in which individuals with milder phenotypes have demonstrated lower QOL [3], from these findings, it appears that caregiver QOL *is* consistent with what has been presumed. It is also of note that while some studies have shown treatment with nusinersen can improve the QOL of patients with SMA 3 in particular [6], this is not consistent with this study with regard to caregiver impact and the type of SMA or ambulatory status.

The QOL subdomains were stratified to better understand the specific impacts of time, emotion and finance on caregivers. Regarding time, there was a trend for the caregivers of individuals with SMA 1 perceiving the most negative impact, followed by SMA 2 and 3. Given that SMA 1 has the highest amount of functional impairment, this trend was expected. In addition, there was a positive influence on time for ambulatory and non-ventilated individuals, trends that were consistent with total QOL score. Similarly, there were no longitudinal changes in time scores for the caregivers of individuals with a given type of SMA or ambulatory status. Surprisingly, the caregivers of ventilated individuals demonstrated improved longitudinal time scores. This information is helpful to consider from the provider standpoint as we aim to respect caregiver needs when scheduling appointments, availability and access to outside support and desires for opportunities for social outlets and vacations.

When considering the subdomain of emotion, it was observed that there was more emotional impact on the caregivers of individuals with SMA 1, followed by SMA 2 and SMA 3. Greater emotional impact was also observed among the caregivers of individuals who were non-ambulatory or required ventilatory support. When looking at changes in emotional impact over time, there were no differences in the caregivers of individuals with different types of SMA, ambulatory status or ventilatory status. This study highlights the emotional burden of caregivers as directly related to individual levels of function and SMA, specifically. This supports the findings of Pacione [7] regarding the emotional impact of treatment and the considerations that families and individuals face when comparing different treatment options. It also highlights the emotional impact of having a neuromuscular disease, regardless of individual functional status, with a greater level of depressive symptoms existing among the parents of children with neuromuscular disorders [9]. There are many elements to consider, including psychological make-up, coping strategies, patient and caregiver temperaments and the presence or absence of daily, social and economic support structures. When stress levels are significantly high, the impact on the physical and psychological health of a caregiver can potentially have adverse effects on the psychological and physical health of the child as well [5]. Connecting caregivers with appropriate support, such as social networks, has been shown to decrease stress levels and positively contribute to the child’s and parent’s resilience. With resilience levels addressed, negative effects resulting from adverse childhood experiences can be decreased or diminished as reductions in family stress and strain have been shown to improve children’s behavioral and emotional strength [17]. 

The subdomain of finance, perhaps the most considered area in the literature, found no differences in financial impact among the caregivers of individuals with different types of SMA or ambulatory status but demonstrated increased impact on the caregivers of individuals requiring ventilatory support. Given the findings of Farrar et al. [6], this may suggest that all individuals with SMA incur certain costs, regardless of their functional level. Over time, there was no change in finance scores among the families of individuals with different SMA types or ambulatory status. However, there was a change over time between the caregivers of those requiring ventilatory support and those who did not, with those not requiring ventilatory support having a slightly decreased rate of change compared to those requiring support. This suggests minimal positive changes for finances with the advent of treatment and may demonstrate increases in costs for both ventilated and non-ventilated children, as incurred through medical appointments, time off from work to receive treatment and the cost of the treatments themselves. These findings regarding the potential for treatment to increase caregiver financial burden over time were also noted by Lee et. al. [8] and are worthy of ongoing consideration from the standpoint of third-party payors. Additionally, consideration should be given to potential changes in household income and possible changes in employment status resulting from the previously noted impacts on time.

There are a few limitations to this study that require further consideration. First, the small sample size, though representative of the varying degrees of function and ventilation requirements, made establishing significance in the data challenging. Future studies should be conducted with larger sample sizes. This study had a relatively limited amount of demographic data as well. Other information, such as feeding status, could offer additional insights, though the perspective of caregivers regarding feeding status is included in the ACEND data. Also of consideration is that the majority of caregivers who completed this survey were mothers. At the outset of this study, we attempted to stratify these groups for more equal representation, but as participants were typically only accompanied by one caregiver, this aspect of the study was not feasible. There was limited caregiver information was collected as well. Insights into caregiver information would be helpful to further investigate key drivers and barriers. Furthermore, the population was limited to a specific geographical area and one neuromuscular center. While the population of caregivers did represent diverse ethnic and socioeconomic backgrounds, multicenter and multisite studies throughout the United States should also be undertaken to fully assess the impact of geography, ethnicity and socioeconomics.

Additionally, at study initiation, individual participants were noted to have variability regarding the initiation of treatment, time on treatment, age and the progression of the disease. Therefore, when considering initial responses and trends, the assessed population became increasingly heterogenous and the consideration of other elements, such as changes in functional status, the need for ventilation and the transition from ambulatory to non-ambulatory, became more important. Future studies should consider looking at age at the initiation of treatment and emerging trends thereof.

All individuals in this study were being treated, therefore, there were no controls or comparisons to ACEND scores for the caregivers of untreated individuals. Additionally, the effects of specific treatments were not compared to define whether one treatment had a greater impact on individual caregiver QOL.

Finally, the specific areas pertaining to QOL were limited in this study to time, emotion and finance. there are likely other areas of impact that need to be taken into consideration as well. Therefore, the findings of this study are limited to the domains measured by the ACEND, which should be considered when drawing conclusions.

## 5. Conclusions

Assessing and addressing QOL concerns for patients and families of individuals with SMA is an integral element in providing comprehensive patient care. Healthcare providers and third-party payors should consider caregiver QOL measures to assess trends as additional markers of treatment effects. As care for individuals with SMA advances, it is important that providers continue to consider caregivers and integrate their QOL concerns by addressing caregiver needs and seeking to ameliorate challenges and stressors for patients and families. Our study showed that the ACEND demonstrated discrimination validity among the caregivers of individuals with different types of SMA and between the caregivers of both ambulatory versus non-ambulatory individuals and between the caregivers of ventilated versus non-ventilated individuals. The subdomains of time and emotion were the most affected by the different levels of functional performance, with the subdomain of finance having minimal differences between groups. For this group of subjects, regardless of SMA type, ambulatory or ventilatory status, there were minimal longitudinal changes in caregiver QOL with the use of disease-modifying treatments. Larger and longer studies are needed to assess whether these trends are statistically significant.

It is possible that even in lieu of advancements in care and improvements in individual function, caregiver quality of life remains unchanged. It is also possible that while the biological needs of the individual are addressed through treatment, the psychosocial and financial needs of the families remain unaddressed, negatively impacted or even ignored. As advancements in care for those with SMA continue to progress, considerations for the QOL of caregivers are of great interest in the hope of informing and continuing to improve the experiences of living with and living for individuals with SMA.

## Figures and Tables

**Table 1:** Summary of participants at baseline.

	Clinical Demographic Domains by SMA Type				Mean Age by Ambulatory Status			Mean Age by Ventilation Status		
**Variable**	**1 (N = 9)**	**2 (N = 13)**	**3 (N = 12)**	**Overall (N = 34)**	**No (N = 27)**	**Yes (N = 7)**	**Overall (N = 34)**	**No (N = 30)**	**Yes (N = 4)**	**Overall (N = 34)**
Age at Baseline										
Mean (SD)	53.28 (58.18)	82.15 (61.29)	95.75 (53.52)	79.31 (58.55)	79.97 (59.48)	76.75 (59.29)	79.31 (58.55)	77.04 (58.73)	96.34 (62.70)	79.31 (58.55)
Median (IQR)	15.62 (7.29, 88.90)	81.72 (32.65, 115.91)	100.40 (51.49, 126.48)	76.04 (24.43, 125.23)	81.72 (18.18, 127.44)	54.94 (33.93, 100.40)	76.04 (24.43, 125.23)	68.50 (24.43, 122.25)	115.50 (69.28, 142.55)	76.04 (24.43, 125.23)
Range	6.63, 143.93	0.36, 185.58	23.66, 190.00	0.36, 190.00	0.36, 185.58	23.66, 190.00	0.36, 190.00	0.36, 190.00	10.42, 143.93	0.36, 190.00
BMI										
Mean (SD)	16.77 (2.10)	19.05 (5.31)	19.45 (5.90)	18.55 (4.87)						
Median (IQR)	17.76 (15.41, 17.89)	16.41 (15.66, 21.59)	18.89 (14.54, 22.46)	17.77 (15.17, 20.06)						
Range	13.00, 19.64	13.61, 31.40	12.37, 31.20	12.37, 31.40						
Missing	0	1	1	2						
**Trache/Ventilation Status**										
No	5	13	12	30						
Yes	4	0	0	4						
**Ambulatory Status**										
No	9	13	5	27						
Yes	0	0	7	7						

**Table 2:** Correlations between Assessment of Caregiver Experience with Neuromuscular Disease (ACEND) scores and scores for the Children’s Hospital of Philadelphia (CHOP), Hammersmith Functional Motor Scale Expanded (HFMSE) and Revised Upper Limb Module (RULM) by SMA type.

**Type 1**			
**Variables**	**Pearson Correlation**	**95% Confidence Interval**	***p*-Value**
Quality of Life by CHOP (Best)	0.51	(0.24, 0.71)	0.0007
Quality of Life by HFMSE	0.60	(0.04, 0.87)	0.0398
Quality of Life by RULM (Total)	−0.15	(−0.77, 0.62)	0.7193
Total ACEND Score by CHOP (Best)	0.80	(0.65, 0.89)	0.0000
Total ACEND Score by HFMSE	0.81	(0.45, 0.95)	0.0013
Total ACEND Score by RULM (Total)	0.44	(−0.38, 0.87)	0.2737
ACEND Time Score by CHOP (Best)	0.70	(0.5, 0.83)	0.0000
ACEND Time Score by HFMSE	0.70	(0.23, 0.9)	0.0083
ACEND Time Score by RULM (Total)	0.13	(−0.63, 0.77)	0.7558
ACEND Emotion Score by CHOP (Best)	0.37	(0.07, 0.61)	0.0160
ACEND Emotion Score by HFMSE	0.61	(0.08, 0.87)	0.0281
ACEND Emotion Score by RULM (Total)	−0.69	(−0.94, 0.03)	0.0584
ACEND Finance Score by CHOP (Best)	0.37	(0.07, 0.61)	0.0176
ACEND Finance Score by HFMSE	−0.02	(−0.56, 0.54)	0.9563
ACEND Finance Score by RULM (Total)	0.52	(−0.3, 0.9)	0.1906
**Type 2**			
**Variables**	**Pearson Correlation**	**95% Confidence Interval**	***p*-Value**
Quality of Life by CHOP (Best)	0.32	(−0.18, 0.7)	0.2032
Quality of Life by HFMSE	0.51	(0.33, 0.65)	0.0000
Quality of Life by RULM (Total)	−0.20	(−0.41, 0.03)	0.0934
Total ACEND Score by CHOP (Best)	0.63	(0.21, 0.85)	0.0067
Total ACEND Score by HFMSE	0.84	(0.76, 0.9)	0.0000
Total ACEND Score by RULM (Total)	0.41	(0.2, 0.59)	0.0003
ACEND Time Score by CHOP (Best)	0.36	(−0.15, 0.72)	0.1552
ACEND Time Score by HFMSE	0.43	(0.24, 0.59)	0.0001
ACEND Time Score by RULM (Total)	−0.07	(−0.3, 0.16)	0.5621
ACEND Emotion Score by CHOP (Best)	0.52	(0.05, 0.8)	0.0335
ACEND Emotion Score by HFMSE	0.55	(0.38, 0.69)	0.0000
ACEND Emotion Score by RULM (Total)	−0.07	(−0.3, 0.16)	0.5517
ACEND Finance Score by CHOP (Best)	−0.31	(−0.69, 0.2)	0.2220
ACEND Finance Score by HFMSE	0.16	(−0.06, 0.36)	0.1614
ACEND Finance Score by RULM (Total)	−0.44	(−0.61, −0.23)	0.0001
**Type 3**			
**Variables**	**Pearson Correlation**	**95% Confidence Interval**	***p*-Value**
Quality of Life by CHOP (Best)	NA	NA	NA
Quality of Life by HFMSE	0.69	(0.54, 0.8)	0.0000
Quality of Life by RULM (Total)	0.56	(0.36, 0.71)	0.0000
Total ACEND Score by CHOP (Best)	NA	NA	NA
Total ACEND Score by HFMSE	0.83	(0.74, 0.89)	0.0000
Total ACEND Score by RULM (Total)	0.66	(0.49, 0.78)	0.0000
ACEND Time Score by CHOP (Best)	NA	NA	NA
ACEND Time Score by HFMSE	0.47	(0.26, 0.63)	0.0000
ACEND Time Score by RULM (Total)	0.32	(0.09, 0.52)	0.0085
ACEND Emotion Score by CHOP (Best)	NA	NA	NA
ACEND Emotion Score by HFMSE	0.63	(0.46, 0.75)	0.0000
ACEND Emotion Score by RULM (Total)	0.46	(0.24, 0.63)	0.0001
ACEND Finance Score by CHOP (Best)	NA	NA	NA
ACEND Finance Score by HFMSE	0.72	(0.58, 0.82)	0.0000
ACEND Finance Score by RULM (Total)	0.67	(0.51, 0.79)	0.0000

Note: CHOP (Best) scores for SMA 3 were all NA, so the correlations for them are all reported as NA.

## Data Availability

The data supporting the findings of this study are available upon request from the corresponding author.
